# Quantitative proteomics reveals that dormancy-related proteins mediate the attenuation in mycobacterium strains

**DOI:** 10.1080/21505594.2021.1965703

**Published:** 2021-10-11

**Authors:** Hong Wang, Li Wan, Jiahui Shi, Tao Zhang, Huiming Zhu, Songhao Jiang, Shuhong Meng, Shujia Wu, Jinshuai Sun, Lei Chang, Liqun Zhang, Kanglin Wan, Jiaqi Yang, Xiuqin Zhao, Haican Liu, Yao Zhang, Erhei Dai, Ping Xu

**Affiliations:** a School of Public Health, North China University of Science and Technology, Tangshan China; b State Key Laboratory of Proteomics, Beijing Proteome Research Center, National Center for Protein Sciences Beijing, Research Unit of Proteomics & Research and Development of New Drug of Chinese Academy of Medical Sciences, Institute of Lifeomics, Beijing, China; c The Fifth Hospital of Shijiazhuang, School of Public Health,North China University of Science and Technology, Shijiazhuang, China; d State Key Laboratory of Infectious Disease Prevention and Control, Collaborative Innovation Center for Diagnosis and Treatment of Infectious Diseases, National Institute for Communicable Disease Control and Prevention, Chinese Center for Disease Control and Prevention, Beijing, China; e The Key Laboratory of Geriatrics,Beijing Hospital, National Center of Gerontology, Chinese Academy of Medical Sciences, Beijing, China; f Key Laboratory of Microbial Diversity Research and Application of Hebei Province, School of Life Sciences and Institute of Life Science and Green Development, Hebei University, Hebei, China; g Department of Biomedicine, School of Medicine, Guizhou University, Guiyang, China; h Department of Biochemistry and Molecular Biology, School of Basic Medical Sciences, Wuhan University, Wuhan, China; i Department of Tuberculosis, Capital Medical University, Beijing Chest Hospital, Beijing, China

**Keywords:** *Mycobacterium tuberculosis*, quantitative proteomics, virulence, attenuation, dormancy

## Abstract

Although members of the *Mycobacterium tuberculosis* complex (MTBC) exhibit high similarity, they are characterized by differences with respect to virulence, immune response, and transmissibility. To understand the virulence of these bacteria and identify potential novel therapeutic targets, we systemically investigated the total cell protein contents of virulent H37Rv, attenuated H37Ra, and avirulent *M. bovis* BCG vaccine strains at the log and stationary phases, based on tandem mass tag (TMT) quantitative proteomics. Data analysis revealed that we obtained deep-coverage protein identification and high quantification. Although 272 genetic variations were reported in H37Ra and H37Rv, they showed very little expression difference in log and stationary phase. Quantitative comparison revealed H37Ra and H37Rv had significantly dysregulation in log phase (227) compared with stationary phase (61). While BCG and H37Rv, and BCG and H37Ra showed notable differences in stationary phase (1171 and 1124) with respect to log phase (381 and 414). In the log phase, similar patterns of protein abundance were observed between H37Ra and BCG, whereas a more similar expression pattern was observed between H37Rv and H37Ra in the stationary phase. Bioinformatic analysis revealed that the upregulated proteins detected for H37Rv and H37Ra in log phase were virulence-related factors. In both log and stationary phases, the dysregulated proteins detected for BCG, which have also been identified as *M. tuberculosis* response proteins under dormancy conditions. We accordingly describe the proteomic profiles of H37Rv, H37Ra, and BCG, which we believe will potentially provide a better understanding of H37Rv pathogenesis, H37Ra attenuation, and BCG immuno protection.

## Introduction

The *Mycobacterium tuberculosis* complex (MTBC) constitutes 11 species [*M. tuberculosis, M. africanum, M. bovis*, Bacillus Calmette-Guérin (BCG), *M. microti, M. canettii, M. caprae, M. pinnipedii, M. suricatta*e, *M. mungi, M. dassie*, and *M. oryx*] that show variable host tropism and virulence [1,2], and share more than 99.95% similarity in gene content and order [1,3,4]. Members of MTBC can cause Tuberculosis (TB) in humans or other animals. In humans, *M. tuberculosis* is the primary etiological agent of TB, and according to the 2019 Global Tuberculosis Report of the World Health Organization (WHO), approximately a quarter of the world’s population is infected with *M. tuberculosis* and at risk of developing TB. In 2018, approximately 10 million new cases of *M. tuberculosis*-related TB were reported, among which there were 1.45 million deaths [5]. Nevertheless, despite concerted global efforts, the mechanisms underlying the persistence, virulence, and pathogenesis of this bacterium remain poorly understood [6].

The characterization of virulent and attenuated strains has been compared to identify potential virulence determinants and gain insights into the pathogenesis and persistence of TB. The attenuated counterpart (H37Ra) of the standard virulent strain H37Rv, obtained by aging H37Rv on solid egg media [7], has been widely used in such studies worldwide. Unlike the virulent strains, H37Ra does not undergo replication in macrophages and resembles the dormant state of *M. tuberculosis* during latent infection [8]. The H37Ra strain also reduced survival under anaerobic conditions [9,10]. Compared to H37Ra, H37Rv infection is characterized by higher bacterial loads in the lungs and other organs [11–13]. Hence, the primary and subsidiary causes of such differences between H37Rv and H37Ra warrant further examination. Comparative genomic analysis have revealed that there several differences in H37Ra that are not present in H37Rv, including 198 single-nucleotide variants (SNVs), 53 insertions, and 21 deletions [14]. For example, the mutation of serine 219 to leucine in the PhoP region of H37Ra [15] affects the PhoP binding to promoter sequences [16,17]. Further, it has also been demonstrated that H37Ra-infected macrophages and mice have higher survival and growth rates in response to the introduction of H37Rv PhoP [16,18]. To date, only the SNV gene of *phoP* (*Rv0757*) has been focused on its potential role in the attenuated H37Ra. There are few reports on the role of the attenuated preprocess of these genetically changing genes.

At present, the only licensed vaccine for the prevention of TB is the BCG vaccine. The BCG vaccine is based on an attenuated strain of *M. bovis*, which was produced by performing 230 serial passages in vitro between 1908 and 1921 by Albert Calmette and Camille Guérin [19,20]. Observational studies have indicated that the BCG strain prevents extrapulmonary TB in children, although in adults, its efficacy against pulmonary disease tends to vary [21,22]. These variable responses in adults are attributed to environmental, demographic, and genetic factors [23] or even the use of different daughter strains [23–25]. Although genome sequencing has indicated that *M. tuberculosis* is highly similar, in terms of gene content and order, to *M. bovis* and various BCG strains [26], 11 regions of difference (RD; 91 genes) of H37Rv are absent from more strains of *M. bovis*, and five additional RDs (38 genes) are only presented in *M. bovis* [27]. Further identification of *M. tuberculosis*-specific [28] or significantly dysregulated proteins could potentially provide a basis for the development of a novel vaccine against TB or diagnosis of this disease.

Although TB-related strains are highly similar, different strains show different extents of host invasion, virulence, and physiological properties. These differences have been investigated to identify virulence determinants based on DNA [14], mRNA [29,30], protein [31–33], post-translational modification (PTM) [34], and macrophage response [35] levels. There has been a considerable amount of attention devoted to understanding the proteomic differences between virulent [H37Rv (virulent), Beijing (strongly virulent strain), or other clinical strains] with attenuated (H37Ra), or virulent (H37Rv) with avirulent (BCG) strains, using two-dimensional polyacrylamide gel electrophoresis (2D-PAGE), label-free, and tandem mass tag (TMT) quantitative analyses. Målen H *et al* used label-free membrane proteomic analyses to identified 19 membrane proteins and lipoproteins that were highly expressed in H37Rv and 10 proteins that were highly expressed in H37Ra, thereby indicating that bacterial secretion and transmembrane transport systems may be important TB-causing determinants [31]. Verma R *et al*. found that compared to H37Ra, the virulence-associated type VII bacterial secretion system was significantly upregulated in H37Rv, and further phosphoproteomic analysis revealed that 84 proteins had different phosphorylation levels [34]. Gunawardena HP *et al*. identified 294 proteins that were differentially expressed between H37Rv and BCG, and these proteins were linked to the lipid and intermediary metabolism, cell wall processes, and transport systems [33]. Bespyatykh J *et al*. used a label-free strategy and detected 266 protein that were different between the Beijing strain B0/W148 and H37Rv, thus, indicating that the B0/W148 strain has higher levels of long-chain fatty acid biosynthesis and lower levels of degradation [36].

To date, there are a limited number of reports on the simultaneous elucidation of broad-scale and precise proteomic analyses using labeled quantitation and high-resolution mass spectrometry (MS) to identify the differences among virulent, attenuated, and avirulent strains. We believe that investigating these differences between the three strain types at the protein level could serve as a basis for gaining insights into the TB-related mechanisms of virulence, attenuation, and immune protection. Accordingly, in this study, we aimed to elucidate the differential protein expression of H37Rv, H37Ra, and BCG during log and stationary phases of growth. To determine differences between protein expression profiles, we used a TMT-labeling strategy combined with high-resolution MS. We identified 3,008 H37Rv proteins, which is one of the largest MS datasets in TB research. Among these, a total of 611 protein groups in 2,463 quantified groups from the log phase and 1,365 protein groups in 3,032 quantified groups from the stationary phase were found to be dysregulated among H37Rv, H37Ra, and BCG. Our large-scale quantification datasets provide a valuable basis for further examination of the differences among virulent, attenuated, and avirulent strains at the protein level, which may in turn contribute to the identification of novel biomarkers and the development of new vaccines.

## Materials and methods

### Strain culture and protein sample preparation

Selected H37Rv, H37Ra, and BCG strains were cultured on 7-mL Lowenstein-Jensen (L-J) slants (Yinke, Zhuhai, Guangdong, China) as described previously [37]. Cell were harvested at the log and stationary phases, and washed three times with chilled phosphate-buffered saline (PBS). To reduce the effects of individual variations, 18 samples of each strain were cultured, and pooling strategies were performed in two quantitative labeling groups. For the log phase, nine biological duplicates were pooled, and the other nine duplicates were used for further verification by TMT-4 labeling. For stationary phase analyses, three duplicate groups of each strain were designed, and six biological samples from each group were pooled for TMT-10 labeling, as previously described [34].

Cells were suspended in a lysis buffer [8 M urea, 10 mM Tris-HCl (pH 8.0), 30 mM NaCl, 10 mM iodoacetamide (IAA), 1× EDTA-free protease inhibitor cocktail)] and disrupted using a FastPrep-24 homogenizer (MP Biomedicals, Santa Ana, CA, USA) with sterile zirconia beads (1.0 mm) for twelve 25-s cycles (30 W). Following centrifugation at 13,000 rpm at 4°C for 20 min, the supernatants were recovered and the total cell protein concentrations were quantified using a previously described gel-assisted method [38]. The gel was imaged and the image was analyzed by Scion Image (4.0.3.2) software (National Institutes of Health, Bethesda, MD, USA).

### Trypsin digestion and TMT labeling

**Figure 1.**
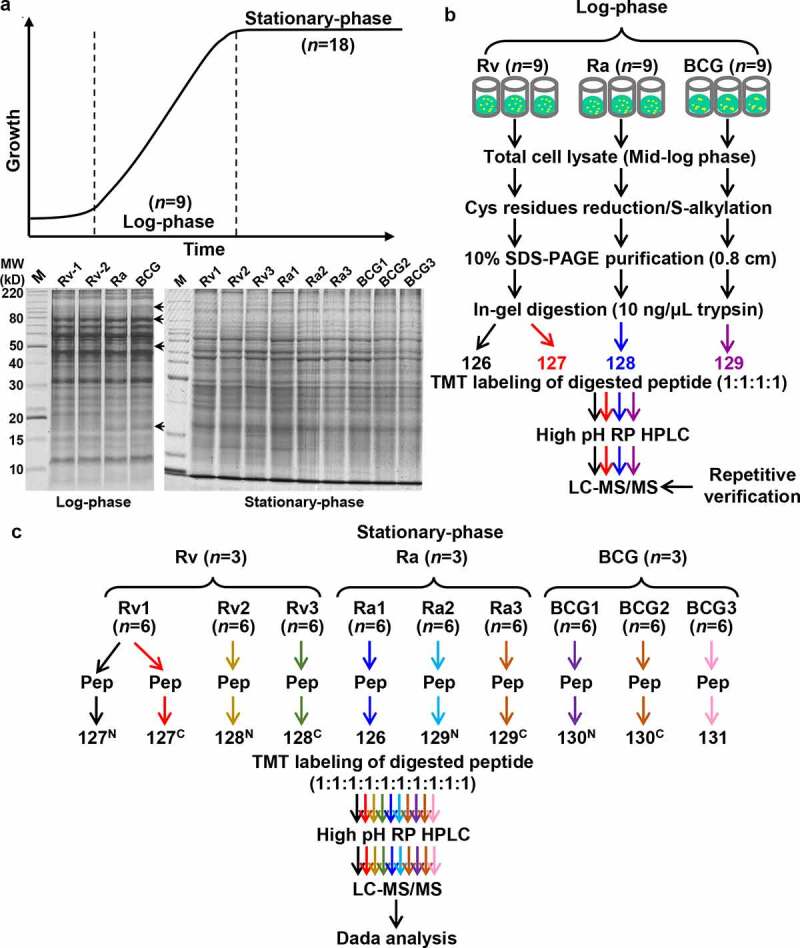
Flowchart for the proteomic profiling

Equal amounts of total cell lysate (120 μg) from each sample were processed, and the technical replicates of H37Rv were considered in our study. These protein samples were reduced with 5 mM dithiothreitol, alkylated with 20 mM of IAA, precleaned with short SDS-PAGE (10%, 0.7 cm), and digested in-gel with 12.5 ng/μL trypsin (Meizhiyuan, Beijing, China) at 37°C for 12 h. Tryptic peptides were labeled with TMT reagents according to the manufacturer’s instructions [34]. In brief, for the log-phase group, H37Rv-1 and H37Rv-2 replicates were labeled with channels 126 and 127, and H37Ra and BCG were labeled with 128 and 129, respectively (Figure 1b). For the stationary-phase group, the H37Rv replicates were labeled with 127 ^N^, 127 ^C^, 128 ^N^, and 128 ^C^; the H37Ra replicates were labeled with 126, 129 ^N^, and 129 ^C^; and the BCG replicates were labeled with 130 ^N^, 130 ^C^, and 131 (Figure 1c). The reaction was quenched by the addition of 8 μL of 5% hydroxylamine, combined, and dried in a vacuum dryer (CentriVap; LABCONCO, Kansas City, MO, USA).

(a) Sample preparation of H37Rv, H37Ra and BCG cultured in the log- and stationary-phase. (b) Overview of TMT labeling quantitation for multiplexed comparative analysis for the log- and (c) stationary-phase samples.

### Two-dimensional liquid chromatography-tandem mass spectrometry (2D LC-MS/MS)

TMT-labeled samples were re-suspended and fractionated using a first-dimensional high pH reverse phase (RP) HPLC system (L-3120; Rigol, Beijing, China) by employing an increasing gradient of buffer B [2% ddH_2_O and 98% acetonitrile (ACN), pH 10] as described previously [38]. Briefly, the solvent gradient of buffer B was held at 0% for 5 min, after which it was incrementally increased from 0% to 3% for 3 min, 3% to 22% for 37 min, 22% to 32% for 10 min, 32% to 90% for 1 min, held at 90% for 2 min, and finally increased to 100% for 2 min. The LC flow rate was set at 0.7 mL/min and monitored at 214 nm, and the column oven temperature was maintained at 45°C. A total of 60 fractions were collected (Figure S1a and 3a) and concatenated into eight (Figure S1b) and 10 (Figure S3b) fractions for the log- and stationary-phase samples, respectively and 10 fractions for phase samples according to the peak capacity. Vacuum-dried fractions were suspended in a loading buffer [0.1% formic acid (FA) and 1% ACN in ddH_2_O] and subjected to LC-MS detection.

The resuspended samples were analyzed using a Q Exactive HF mass spectrometer (Thermo Fisher Scientific, Waltham, MA, USA). Briefly, samples were loaded onto a self-packed capillary column (75 μm i.d. × 50 cm, 1.9 μm C_18_) and eluted with a 135-min linear gradient, in which solvent B was incrementally increased as follows: 4% to 8% for 13 min, 8% to 25% for 86 min, 25% to 50% for 21 min, 50% to 90% for 3 min, and held at 90% for 12 min. Full MS scans were performed over an *m/z* range of 375 to 1,400 at a resolution of 1.2 × 10^5^. The maximum injection time (MIT) was set to 80 ms, and the automatic gain control (AGC) was set to 3.0 × 10^6^. For MS/MS scans, the 15 most intense peptide ions with charge states of 2 to 6 were subjected to fragmentation via higher energy collision-induced dissociation (AGC, 1 × 10^5^; MIT, 45 ms; Resolution, 3.0 × 10^4^). Dynamic exclusion was set at 30 s.

### Database searches for peptide identification and quantification

The acquired raw files were searched against a composite target/decoy database using MaxQuant (v1.5.5.1) to determine the false discovery rate (FDR). The protein databases used for MS/MS searches for H37Rv, H37Ra, and BCG were downloaded from NCBI (1 February 2013), NCBI (14 March 2017), and NCBI (updated 15 May 2017), and included 3,912, 4,278, and 4,224 protein sequences, respectively. A combined database consisting of the same proteins and unique proteins from H37Rv, H37Ra, and BCG (containing 5,596 entries), along with 245 common contaminant protein sequences (http://www.maxquant.org) were obtained.

The search parameters included trypsin as a proteolytic enzyme, with two missed cleavages allowed. Oxidation of methionine was set as a dynamic modification, whereas carbamidomethylation of cysteine and TMT modification at the peptide N-terminus and lysine were set as fixed modifications. The peptides and proteins were filtered to an FDR 1% using a target–decoy search strategy.

For each sample, the quantified values in the file proteinGroups were ranked, and the mean intensity was normalized. Significantly different proteins were identified using an in-house script that was based on the “significance B” theory approach with Benjamini−Hochberg FDR of < 5% for log-phase quantitative datasets [39], and *T-test* analysis was performed for stationary-phase quantitative datasets using the Perseus (v1.6.6.0) software to generate heat maps [40].

The technical variability was evaluated, using the intensity ratio of the H37Rv sample labeled with 126 and 127 tags for the log phase, and 127 ^N^ and 127 ^C^ for the stationary phase, based on the respective correlation coefficient (R^2^) values and standard deviation (SD). Dysregulated proteins were selected based on 5× SD values and a *p-value* 0.05 for H37Ra versus H37Rv, BCG versus H37Rv, and BCG versus H37Ra. The regulated proteins obtained were used for further bioinformatics analyses.

### Bioinformatic analysis

Differentially expressed proteins (DEPs) that were identified in this study were subjected to functional analysis using the DAVID online platform, including enriched pathways, biological process (BP), and molecular function (MF) analyses [41]. Protein interaction analysis was conducted using the STRING database [42].

The mass spectrometry proteomics data were deposited in the ProteomeXchange Consortium database (http://proteomecentral.proteomexchange.org) via the PRIDE partner repository with the dataset identifiers PXD017174 and PXD023788.

## Results and discussion

### H37Rv, H37Ra, and BCG show similar protein patterns

For the present study, we used H37Rv, H37Ra, and BCG as representative virulent, attenuated, and avirulent TB strains, respectively. To determine differences between the protein expression patterns among these strains, whole-cell lysates were separated using 10% SDS-PAGE. Differences in the proteins were observed at 105-, 80-, 50-, and 17-kDa in the log-phase samples (Figure 1a). No notable differences were observed across the stationary-phase samples, which tended to show similar patterns. We obtained clearly defined bands for proteins from high to low molecular weight in each lane, thereby indicating that we had prepared high-quality total cell lysates with similar concentrations. Next, we identified the regulated proteins and signaling networks that were potentially associated with virulence or immunity. A comparative proteomic analysis of H37Rv, H37Ra, and BCG using a TMT-labeling-based quantitative strategy was performed (Figure 1b and 1c). The proteomic profiles of the three strains, harvested at log and stationary phases from colonies cultured on L-J solid media slants, were analyzed using LC-MS/MS. To assess technical variation, we used duplicates by splitting the same extracted proteins of H37Rv into two identical portions, which enabled us to determine the variation introduced during in-gel purification, digestion, labeling, and subsequent analysis. To eliminate individual variation, we prepared 18 biological replicates of the cultures and adopted a pooling strategy in two labeling experiments.

### Proteomic profiles of H37Rv, H37Ra, and BCG in the log and stationary phases

**Figure 2.**
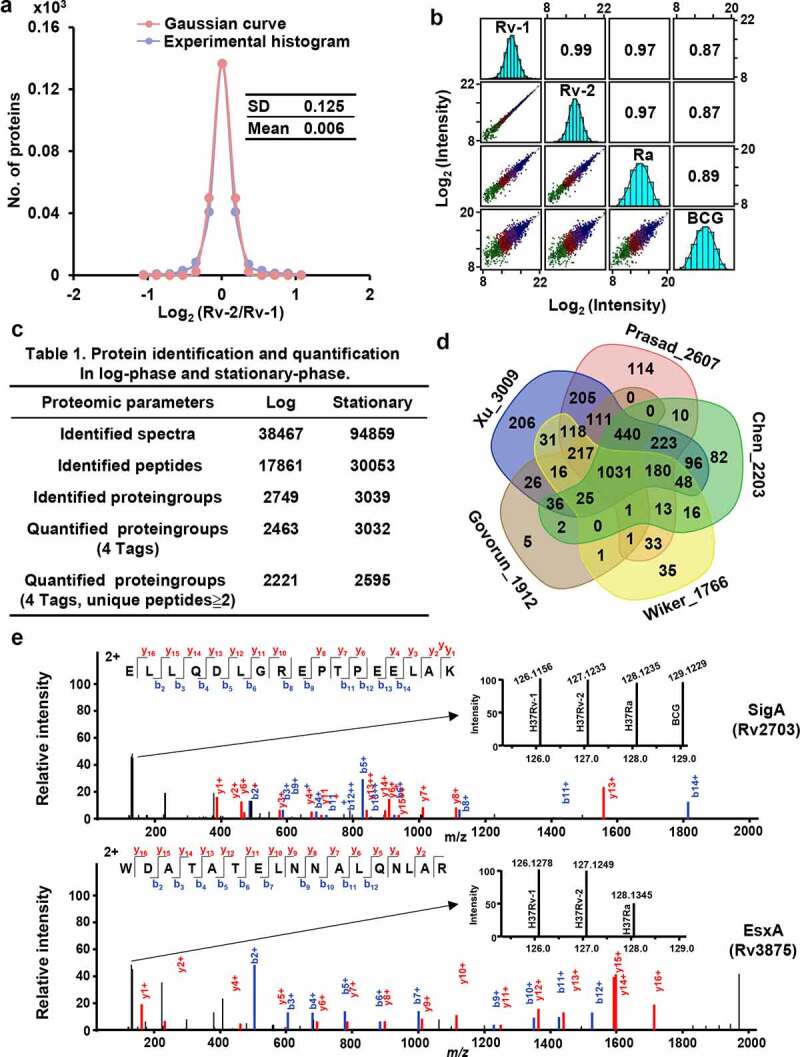
Large-scale quantitative proteomics for H37Rv, H37Ra, and BCG in log-phase

We detected no obvious difference between the technical repeats that were performed for the log- and stationary-phase samples. For H37Rv replicates, the SD values were 0.125, 0.03, and 0.15, respectively (Figure 2a, S2a, and S4a), and the corresponding R^2^ values of 0.99 and 1.00 (Figure 2b, S2b, and S4b) indicate high reproducibility of the results and that the experimental and verification procedures were well-controlled.

(a) The Gaussian fitting curve of log_2_ ratio of the intensities of technical replicates. Red and blue curves represent the Gaussian and experimental fitting curves, respectively. (b) Scatter plots and Spearman’s correlation coefficients for proteome profiling of four samples. The *x* and *y* axes represent the log_2_-transformed protein intensities in each two-sample comparison. (c) Summary of MS identification and quantitation. (d) Comparison of the number of quantified proteins in this study with recent literatures. (e) The quantitative values of the RNA polymerase sigma factor sigA (Rv2703) and the H37Rv RD-1 protein esxA (Rv3875) among four samples.

In the log phase, we identified 17,861 peptides corresponding to 2,749 protein groups (Table S1), of which 2,463 proteins were simultaneously quantified by four tags. Among the quantified proteins, 2,221 protein groups (90.17%) that contained more than one unique peptide were identified (Figure 2c). Comparatively, in the stationary phase, we identified a higher number of peptides (30,053) and protein groups (3,039), which could be attributed to the larger number of samples and concatenated fractions. Among the identified proteins, 86.07% of H37Rv annotated proteins were commonly detected in log and stationary phase samples, and 99 (3.29%) and 320 (10.64%) proteins were uniquely identified in log and stationary phase, respectively (Figure S5a). The uniquely identified proteins in the log phase were significantly enriched in arsenite transport [ArsC (Rv2643), ArsA (Rv2684), and ArsB1 (Rv2685)], growth of symbiont in host cell [Rv0325, ArfB (Rv0900), VapC (Rv2548), Rv3371, and LipF (Rv3487c)], anaerobic respiration [FrdA (Rv1552) and FrdB (Rv1553)] and cytochrome complex assembly [CcdA (Rv0527) and DipZ (Rv2874)] (Figure S5b). The uniquely identified proteins in the stationary phase were related to virulence proteins PPEs [PPE14 (Rv0915c), PPE26 (Rv1789), PPE41 (Rv2430c), PPE45 (Rv2892c), and PPE50 (Rv3135)], secretory proteins [EccE3 (Rv0292), EsxS (Rv3020c), and EsxC (Rv3890c)], MMPSs [MmpS2 (Rv0506) and MmPS4 (Rv0451c)] (Figure S5c).

The identified H37Rv proteins (3,008) were compared to those obtained from previously published *M. tuberculosis* proteomic studies. The comparison revealed that the dataset compiled in the present study is the largest dataset obtained to date, which included approximately three-quarters of the total annotated H37Rv proteins in the TubercuList database (4,032). We identified 206 unique proteins, indicating that more than 93.15% of proteins detected in the present study were also included in other H37Rv proteomic datasets (Figure 2d).

The H37Rv and H37Ra strains were derived from the same parent H37 strain, which was reflected in the high R^2^ values obtained in the three independent experiments. The BCG strain is an avirulent *M. bovis* strain and expectedly showed a lower R^2^ value, given the distant phylogenetic relationship between *M. tuberculosis* and *M. bovis*. These results indicate that a greater difference in the virulence among the H37Rv, H37Ra, and BCG is reflected by a higher proportion of DEPs. Accordingly, the expression characteristics of dysregulated proteins in these three strains during different growing phases will potentially contribute to clarifying their pathogenicity and immune-protective properties.

### Differences in the expression profiles of H37Rv, H37Ra, and BCG during the log phase

To validate the accuracy of our quantified values, we examined the expression of housekeeping genes among H37Rv, H37Ra, and BCG and the genes that have been deleted in the BCG strain. In this regard, *sigA* (*Rv2703*) is a primary cytoplasmic sigma factor of *Mycobacterium* that controls the transcription of the housekeeping type of promoters and is considered a good candidate for internal control of total RNA or protein expression [43,44]. Chen *et al*. found no differences between the SigA levels among membrane proteins of H37Rv and BCG using label-free and western blot analyses [33]. In the present study, we found that the quantitative values of SigA obtained for the H37Rv, H37Ra, and BCG strains were virtually identical (1:0.96:0.97), thereby implying that our quantification of the proteins in these strains was reasonably accurate (Figure 2e). BCG is an attenuated *M. bovis* strain that is characterized by a loss of 16 RDs of differing lengths [45]. Among these deleted regions, RD1 contains *esxA* (*Rv3875*), which is a typical virulence factor that is often used as a clinical diagnostic marker of *M. tuberculosis* infection and is regarded as a good candidate for vaccine development [46]. The quantitative data we obtained indicated that the EsxA protein was lost in the BCG, and was also found to be downregulated (0.45) in H37Ra. Accordingly, we believe that our accurately quantified datasets will provide a good opportunity to further characterize the differences among of three assessed strains with respect to virulence.

**Figure 3.**
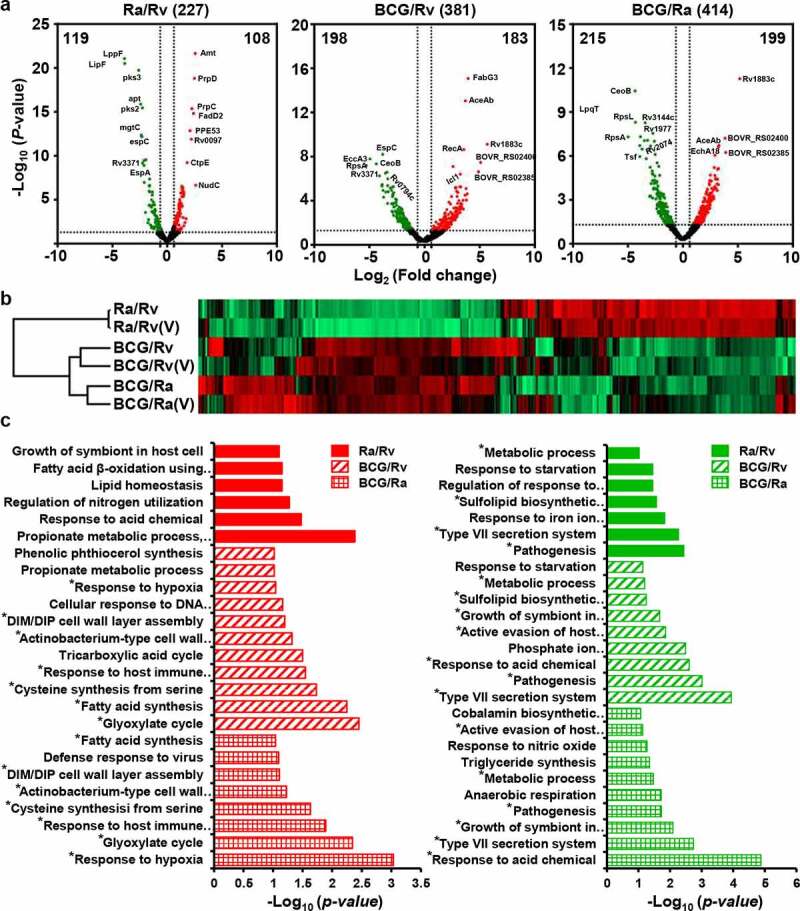
Overall differentially expressed proteins among H37Rv, H37Ra, and BCG in log-phase

A total of 2,463 quantified proteins were selected for differential expression analysis, based on the criteria of SD values > 5 or < −5 [fold change > 1.542 or < 0.648] and *p* < 0.05. To profile the expression levels of these DEPs, we used GraphPad Prism (v5) to construct volcano plots [47], which illustrated that the greater the difference in the virulence of the strains, the higher is the number of differentially expressed proteins, and these proteins tended to show greater fold changes (Figure 3a). Following stringent filtering, we selected a total of 611 DEPs, among which 227, 381, and 414 were differentially expressed in the H37Ra versus H37Rv, BCG versus H37Rv, and BCG versus H37Ra groups, respectively (Table S2). The expression of these DEPs was verified by performing repeat experiments (Figure 3b, Tables S3 and S4). We found that 86.70% of the proteins were differentially expressed between H37Ra and H37Rv. The dysregulation was consistent with at least one of our verified datasets (Wiker *et al* [48]., Prasad *et al* [34].) (Table S6), and 96.05% of DEPs from the BCG and H37Rv groups were also shown to be dysregulated in at least one of our verified datasets (Gordon *et al* [49]., Chen *et al* [33].) (Table S7). These findings validated our selection of DEPs, which comprise of the first large-scale lists of proteins that are differentially expressed among H37Rv, H37Ra, and BCG based on quantitative labeling methods, and represent a valuable resource for follow-up functional processing.

(a) Volcano plots of the DEPs between H37Ra and H37Rv, BCG and H37Rv, and BCG and H37Ra. Red and green in the log-phase green dots represent upregulated and downregulated proteins, respectively. (b) Comparison of the DEPs from the log-phase between two different duplication experiments. V stands for verification experiment. (c) BP enriching analysis of the DEPs from H37Ra and H37Rv (picture fill), BCG and H37Rv (tilt texture), and BCG and H37Ra (box texture), respectively. Red and green represent upregulated and downregulated proteins, respectively. * stands for repeated emergence

*Log phase virulence factors and stress-responding proteins regulate virulence processes, whereas dormancyrelated proteins mediate attenuation programs*

We observed two typical expression patterns for BCG/H37Rv and BCG/H37Ra in the heat map, which were consistently downregulated or upregulated (Figure S6a). BP enrichment analysis of the DEPs from these two clusters revealed that the downregulated proteins (174) were primarily enriched in the following functional categories: type VII secretion system (similar to Prasad *et al* [34].), pathogenesis, active evasion of host immune response, cobalamin biosynthetic process, growth of symbiont in host cells, response to acid chemical, and sulfolipid biosynthesis. *M. tuberculosis* can evade the host immune system by virtue of its tolerance to certain adverse conditions, including O_2_ depletion, nutrient limitation, NO, and acid stimulation. We found that compared with BCG, both H37Rv and H37Ra showed higher expression of virulence factor-related proteins (secreted protein, PE/PPE, lipoprotein, and sulfolipid biosynthesis proteins) and stress-response proteins, including those associated with responses to starvation and acid. These upregulated proteins (176) were related to hypoxia response, fatty acid biosynthetic process, response to host immune response, tricarboxylic acid cycle, actinobacterium-type cell wall biogenesis, DIM/DIP cell wall layer assembly, and cellular response to DNA damage (Figure S6b, Figure 3c, and Figure 7).

**Figure 4.**
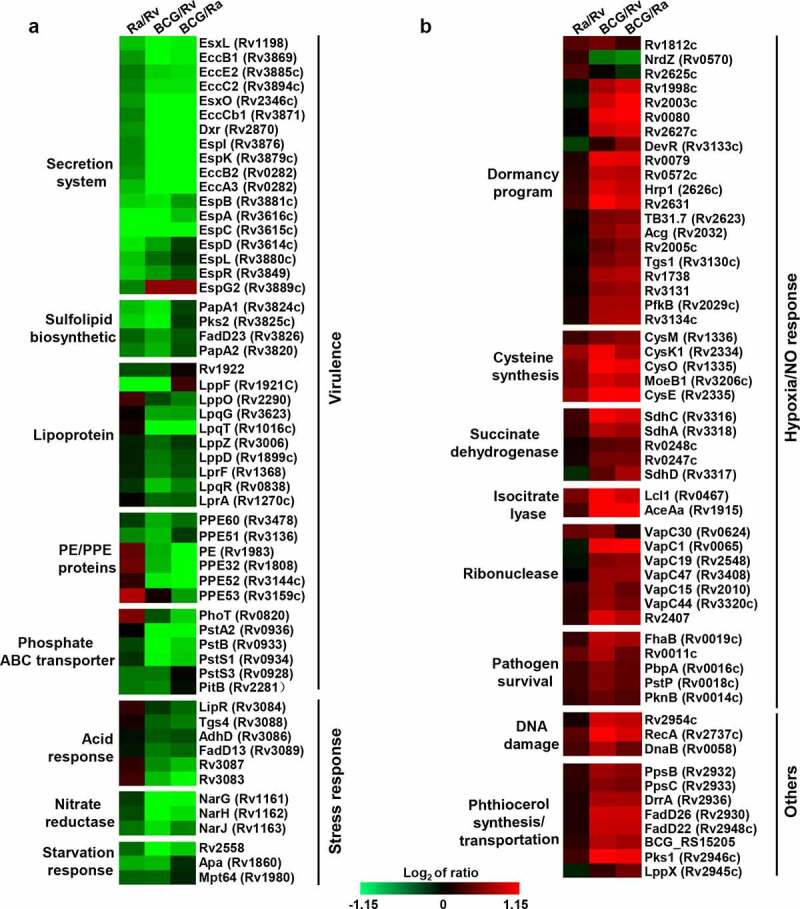
Two typical expression models between BCG and H37Rv, and BCG and H37Ra groups in the log-phase

(a)The consistent downregulating and (b) upregulating profiles observed for BCG and H37Rv, and BCG and H37Ra groups, respectively.

**Figure 5.**
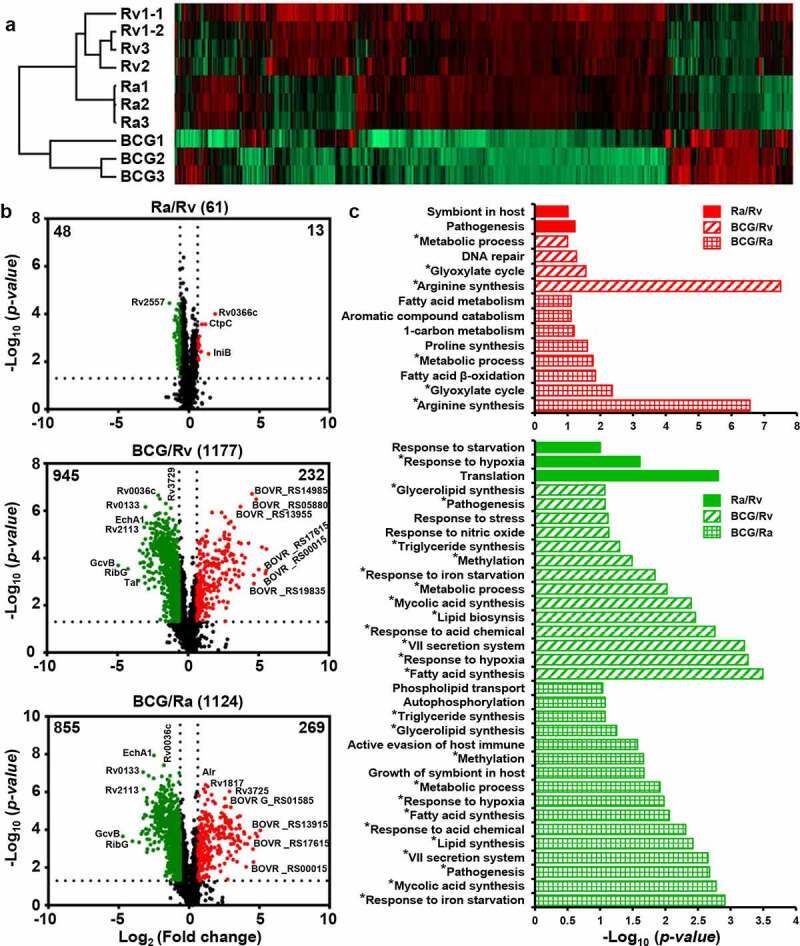
Large-scale quantitative proteomics among H37Rv, H37Ra, and BCG in the stationary-phase

(a) The global protein expression map in the stationary-phase, including technical and biological duplications. (b)Volcano plots of the DEPs between H37Ra and H37Rv, BCG and H37Rv, and BCG and H37Ra. (c) BP enriching analysis of the differentially expressed proteins from H37Ra and H37Rv, BCG and H37Rv, and BCG and H37Ra groups, respectively.

**Figure 6.**
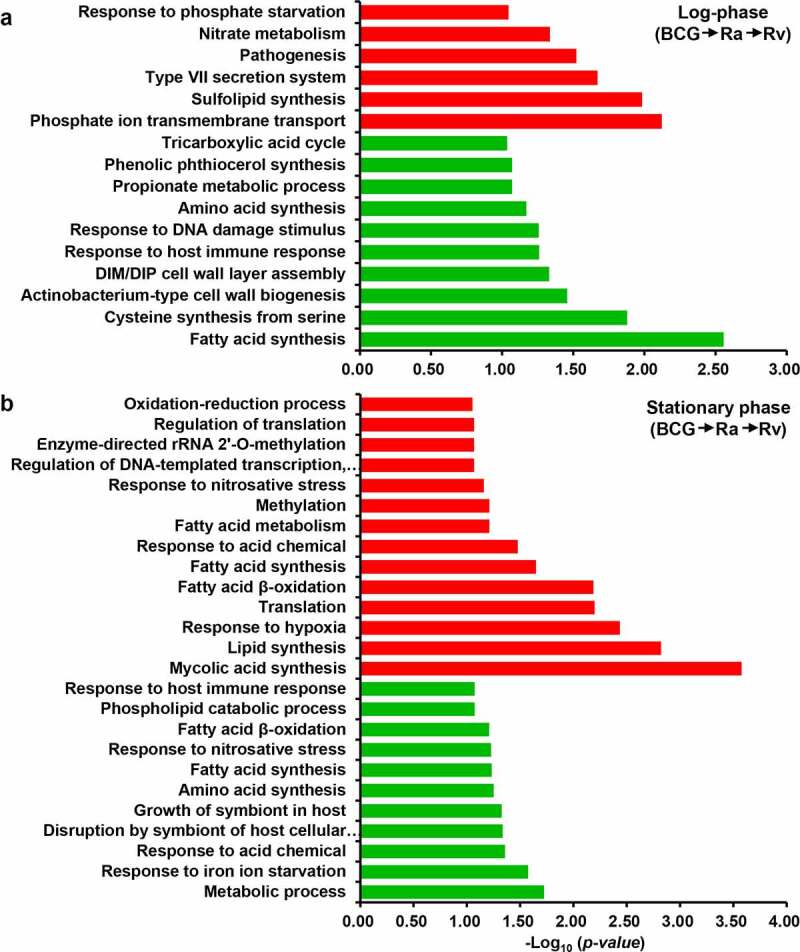
Comparison of BP enrichment of DEPs with increasing virulence between (a) log- and (b) stationary-phase

**Figure 7.**
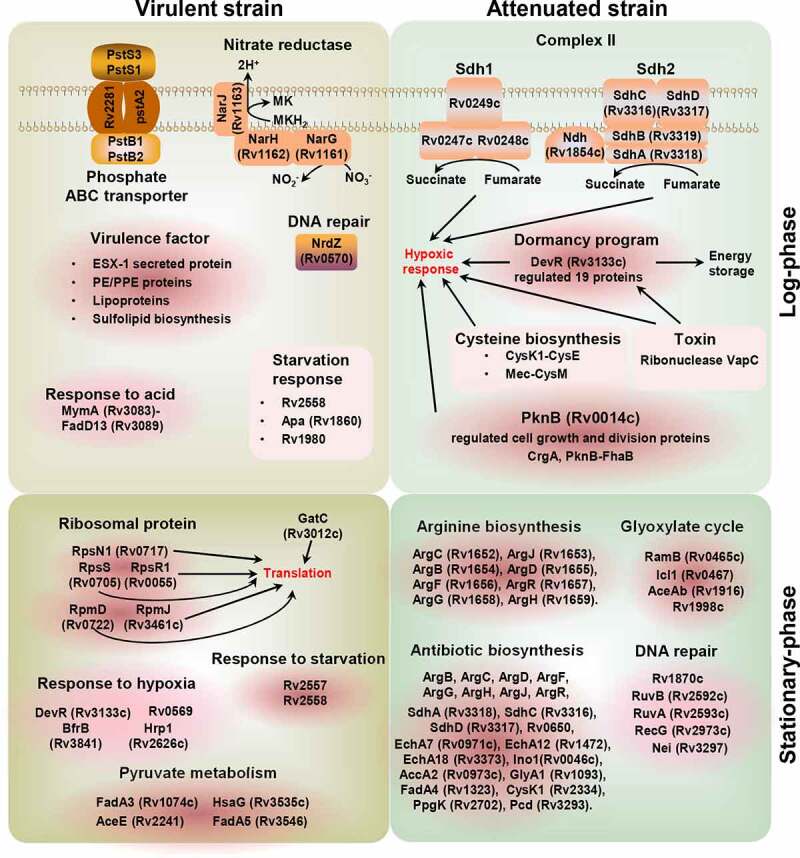
Schematic model for pathogenic and immune-protection effects caused by the virulent and attenuated strains in the log- and stationary-phase

Further functional classification revealed that virulence-related DEPs were associated with secretion, lipid synthesis, PE/PPE, phosphate ABC transporter, acid and starvation response, and nitrate reductase proteins (Figure 4a), which may be positively regulated by the transcriptional regulators EspR (Rv3849), DevR(Rv3133c), TcrX (Rv3765c), and PtkA (Rv2232) (Figure S7A). Among the secretion system-related DEPs, EspD (Rv3614c)–EspA (Rv3616c), which together form an operon, are involved in the ESX-1 secretion system required for Snm (secretion in mycobacteria) and are essential virulence determinants in *M. tuberculosis* [50–53]. It has previously been found that EspC (Rv3615c) is a highly immunodominant RD1-dependent secreted antigen that is specific to *M. tuberculosis* infection [51]. The secreted DEPs identified in the present study may have implications for TB diagnosis and vaccine development. Sulfolipid-1 (SL-1), the most abundant sulfatide that is uniquely expressed in pathogenic mycobacteria, is located exclusively in the outer membrane of *M. tuberculosis*, and its levels have been positively correlated with strain virulence [54,55]. PapA1 (Rv3824c)–FadD23 (Rv3826), which was found to be significantly upregulated in H37Rv, is essential for the biosynthesis of SL-1, and could represent a potential drug target.

During bacterial growth, there is a gradual decrease in oxygen and nutrients, and it has been found that the transition to hypoxic conditions in *M. tuberculosis* is associated with the upregulation of specific enzymes, including nitrate reductase [56]. The expression of the *NarGHJI* (Rv1161–Rv1163) operon is upregulated in both virulent H37Rv and attenuated H37Ra. This indicates that compared with BCG, strains with a higher level of virulence could use nitrate as a final electron acceptor for the maintenance of a proton motive gradient to facilitate continued growth [56,57]. A notable feature of *M. tuberculosis* is its ability to survive and proliferate within host macrophages, thereby inhibiting the acidification and maturation of phagosomes. This ability is associated with the expression of the acid-induced operon MymA (Rv3083)–FadD13 (Rv3089) [58], which is consistently upregulated in the virulent strains H37Rv and H37Ra at the protein level. Additionally, the starvation response-related proteins, including subunits of the phosphate-specific transporter (Pst) [PstS3 (Rv0928), PstS1 (Rv0934), PstA (pstA2, Rv0936), PstB [PstB1 (Rv0820), and PstB2 (Rv0933)], and three other proteins [Rv2558, Apa (Rv1860), and Mpt64 (Rv1980)], have also been found to be upregulated in virulent strains. It has also been established that certain Pst subunits are important for the survival of *M. tuberculosis* in nutrient-deficient environments, particularly under conditions of Pi starvation [59], and macrophage infection [60,61]. Furthermore, the expression of the vitamin B_12_-dependent ribonucleoside-diphosphate reductase NrdZ (Rv0570) is upregulated in virulent strains, indicating that a pool of deoxyribonucleotides is required to maintain chromosomal integrity or complete DNA repair processes [54].

With respect to the BCG strain, the detected DEPs were associated with dormancy, cysteine synthesis, succinate dehydrogenase, isocitrate lyase, ribonuclease, pathogen survival, DNA damage, phthiocerol synthesis, and transportation proteins (Figure 4b, and Figure 7), and may be positively regulated by the transcriptional regulators DevR (Rv3133c), Mce1R (Rv0165c) and Rv2989, RNA polymerase sigma factor SigB (Rv2710), and the regulatory protein RecX (Rv2736c) (Figure S7a-c). Among these is DosR, a classical response regulator of the two-component class, which regulates a set of 47 *M. tuberculosis* dormancy genes in response to hypoxia and NO [60,61]. We found that DosR and 18 dormancy-related proteins were significantly upregulated in the BCG strain, and, consistent with previous respiration regulation studies, we also identified further three anaerobic response groups that were upregulated in BCG, namely, two independent cysteine synthesis pathways [CysK1 (Rv2334)-CysE (Rv2335) and CysO (Rv1335)-CysM (Rv1336)] [62–64], succinate dehydrogenase [Sdh1 (Rv0247c and Rv0248c), Sdh2 (sdhC-sdhA)] [65], and isocitrate lyase [Icl (Rv0467) and AceAa (Rv1915)][66]. SigB is expressed in the exponential phase, and induced by low aeration (2.5-fold) and oxidative stress (2.7-fold; SigE and SigH responsive) [67–70]. Under conditions of nutrient starvation [71], members of toxin–antitoxin systems were expressed at higher levels in the BCG strain, which is assumed to reflect the role played by these modules during the period of metabolic transition, whereby the BCG strain enters a dormancy-like state in which growth is negatively regulated. Upon reaching the log phase in sealed L-J slants, BCG may gradually undergo a transition to a non-replicating persistence (NRP) stage, which is characterized by a dormancy regulon containing 20 genes, dormancy-regulated toxin genes [VapC1 (Rv0065), VapC30 (Rv0624), VapC15 (Rv2010), VapC19 (Rv2548), VapC44 (Rv3320c), and VapC47 (Rv3408)]; hypoxic response protein groups including succinate dehydrogenase, and cysteine biosynthesis; and cell growth and division proteins (Figure S8). The enriched biological processes for the proteins that were differentially upregulated or downregulated between BCG and H37Ra were almost entirely similarly differentially expressed between BCG and H37Rv, with the few exceptions being proteins associated with the defense response to virus, anaerobic respiration, and cobalamin biosynthesis.

*In the stationary phase, the H37Rv and H37Ra strains show similar levels of protein expression, whereas the BCG strain shows significant differences*

Our data revealed that in the stationary phase, similar relative levels of protein expression were observed across the H37Rv1 technical duplicates and biological duplicates for each of the three strains (Figure 5a). In the log phase, the BCG strain showed certain differences in protein expression compared with the H37Rv and H37Ra strains, the extent of differences was notably more pronounced in the stationary phase, whereas comparatively fewer differences were detected between H37Ra and H37Rv at this stage (Figure 5b). In total, we identified 1,365 proteins that were differentially expressed, among which 61, 1,177, and 1,124 were found to differ between H37Ra and H37Rv, BCG and H37Rv, and BCG and H37Ra, respectively (Figure 5b and Table S10). Compared with H37Rv and H37Ra, more than 20% of proteins were dysregulated in BCG during the stationary phase, and BP analysis revealed that the DEPs detected in the BCG strain were primarily enriched in arginine synthesis, glyoxylate cycle, and fatty acid metabolism (Table S11). In this regard, Jacobs *et al* [72]. demonstrated that the de novo arginine biosynthetic pathway in *M. tuberculosis* is upregulated during early responses to oxidative stress. In the present study, we found that eight arginine synthesis proteins were significantly upregulated in the BCG strain, namely, ArgC (Rv1652), ArgJ (Rv1653), ArgB (Rv1654), ArgD (Rv1655), ArgF (Rv1656), ArgR (Rv1657), ArgG (Rv1658), and ArgH (Rv1659), which may indicate that the H37Rv and H37Ra strains experience a deficiency in arginine during the stationary phase. The glyoxylate cycle is an alternative pathway used to generate energy when the tricarboxylic acid cycle is limited by oxygen and nutrient depletion [73]. Several plants and microorganisms, particularly *M. tuberculosis*, are dependent on glyoxylate cycle enzymes to survive under circumstances in which components of the tricarboxylic acid cycle are downregulated. We found that four glyoxylate-related enzymes were upregulated in the BCG strain, namely, Icl1 (Rv0467), AceAb (Rv1916), GlcB (Rv1837c), and Rv1998c; among these, McKinney *et al*. previously demonstrated that during the downregulation of the tricarboxylic acid cycle, inhibition of *icl1* is fatal for *M. tuberculosis*. RamB (Rv0465c), an HTH-type transcriptional regulator, is involved in the control of the glyoxylate cycle, which may be positively regulated by the HTH-type transcriptional regulator RamB (Figure S8) and could be considered a potential drug target for *M. tuberculosis* during dormancy. The other regulated HTH-type transcriptional regulator Rv0081 is a member of the dormancy regulon induced in response to hypoxia, low NO, and CO [74–77], which will provide insight into the latent or dormant phase of infection.

DEPs with downregulated expression during the stationary phase were primarily enriched in processes associated with fatty acid, mycolic acid, and lipid synthesis, type VII secretion system, pathogenesis, response to various stress conditions (hypoxia, starvation, and NO), growth of symbionts in hosts, and active evasion of host immunity. In this regard, it is well established that during persistent infection in macrophages, mouse challenge models, and within human lung tissues, *M. tuberculosis* uses alternative metabolic pathways to consume fatty acids instead of carbohydrates [64,78–81]. We found that in the BCG strain, 25 proteins associated with fatty acid synthesis were consistently downregulated, thereby implying that fatty acid metabolism may influence host growth and virulence. In addition, it is conceivable that the preferential synthesis of lipids by *M. tuberculosis* is associated with the pathology [82]. Mycolic acid, which has been detected in samples of patient sputum and comprises a large portion of the mycobacterial cell wall [83,84], represents an ideal therapeutic target. We found that lipid- and mycolic acid- synthesizing proteins were expressed at significantly lower levels in the BCG strain, indicating that these could be virulence-related factors for *M. tuberculosis*. Stress-related DEPs detected in the BCG strain, which differed from those characterized during the log phase, were significantly downregulated in the stationary phase. These included stress response proteins [MprA(Rv0981), Rv1996, Rv2005c, Rv2026c, Rv2028c, Rv2035, TB31.7 (Rv2623), Rv2624c, and Rv3134c], NO DEPs [Rv0221, Rv1425, Rv2285, Rv3087, Tgs4 (Rv3088), Tgs1 (Rv3130c), Rv3480c, and Tgs2 (Rv3734c)], acid chemical [PrpD (Rv1130), Rv3083, LipR (Rv3084), Rv3085, AdhD (Rv3086), Rv3087, Tgs4, FadD13 (Rv3089), NarG (Rv1161)], iron ion DEPs [MbtA (Rv2384), HupB (Rv2986c), EccA3 (Rv0282), Rv0560c, Rv3402c, IrtA (Rv1348), IrtB (Rv1349), MbtB (Rv2383c), MbtA(Rv2384), MbtI (Rv2386c), and Pks2(Rv3825c)], and 25 hypoxia DEPs.

*Virulence factors are continuously upregulated in the log phase, whereas dormancy-similar characterized proteins are observed in the stationary phase*

The oxygen-depleted dormancy model is commonly referred to as the NRP model or “Wayne” model [85]. Schoolnik *et al*. found that during the stationary phase, *M. tuberculosis* may continue to replicate at a low level as opposed to surviving in a state of NRP [86]. Comparative proteomic analysis revealed that H37Rv expressed the highest levels of virulence factor-related proteins during the log phase compared with the stationary phase. These proteins were associated with phosphate transport, sulfolipid synthesis, pathogenesis, and nitrate metabolism (Figure 6a and b), thereby indicating that during log phase, H37Rv maintains good growth status, which results in its strong pathogenicity. Contrastingly, the BCG strain showed a very similar dormancy phenomenon in both the log and stationary phases, given that the detected DEPs in this strain were associated with metabolic shifts (such as, carbon flux [87], lipid metabolism of the *M. tuberculosis* cell wall [88,89], and fatty acid metabolism), and processes related to external stimuli (such as, hypoxia, NO, nutrient starvation, deficiency of metal ions, and low pH). Although H37Rv is also characterized by periods of dormancy with features similar to those observed in the stationary phase, we found that 20 translations and 3 translation regulatory proteins were upregulated. Virulent *M. tuberculosis* has a more rapid in vivo doubling time and is better equipped to resist growth-inhibiting macrophages [90]. Moreover, viable H37Rv and H37Ra can escape from fused vesicles with the progression of infection, whereas the BCG strain lacks this capacity [8]. Accordingly, given the findings of these in vivo studies, it is likely that compared with the BCG strain, H37Rv has a greater capacity to continue growth during the stationary phase.

*Genetic changing genes detected in H37Ra compared to H37Rv show fewer dysregulation in the log phase but not in the stationary phase*

Compared with H37Rv, 53 insertions, 21 deletions, and 198 SNVs were identified in H37Ra [91,92]. In the log-phase, only one inserting gene HadC (Rv0637, 0.33), two SNV proteins [Rv1021 (0.37) and Rv2037c (0.60)] were downregulated in H37Ra compared with H37Rv (Table S2). These genetic differences did not result in differential expression between H37Rv and H37Ra in the stationary-phase (Table S10). Expression analysis of gene variants implies that they may play a minor role in viral attenuation at the whole-cell level in comparison to the DEPs (virulence factors, dormancy, etc.) that were detected in this study by large-scale quantitative proteomics.

## Conclusions

In summary, the quantitative data obtained from this study indicated that broad-scale proteomic features of virulent H37Rv, attenuated H37Ra, and avirulent BCG. These differences are likely to underpin the different virulence characteristics of these strains. The H37Ra genome differs from that of H37Rv because of 53 insertions, 21 deletions, and 198 SNVs. Quantitative analysis revealed that these genetic variations had very little differences in the expression profiles between H37Ra and H37Rv in the stationary phase. Compared with genetic variations’ expression, we detected notable differences (227) in the protein expression profiles of H37Rv and H37Ra during the log phase of growth, and a small number (61) of dys-regulating proteins in the stationary phase. The virulent, stress-responding, and translating DEPs were significantly upregulated in the virulent strain H37Rv, which may be positively regulated by the transcriptional regulators EspR, DevR, TcrX, and PtkA (Figure S7a). With respect to the BCG strain, compared with the log phase, a larger number (>1000) of proteins were found to be dysregulated in the stationary phase, thereby indicating that this avirulent strain is characterized a more pronounced difference in the expression of proteins in the slowly replicating stages than either H37Rv or H37Ra. Furthermore, in the case of both BCG and H37Ra, previously reported *M. tuberculosis* dormancy-related proteins were upregulated in the log phase, but they were downregulated in the stationary phase. These results indicate that these DEPs positively and negatively regulate the attenuation process in log and stationary phases, respectively. The findings of this comparative proteomic analysis will contribute to enhancing our current knowledge of tuberculosis-related strains, shedding new light on the characteristics of virulent and attenuated strains. This improved understanding of TB biology can provide valuable insights for the design of diagnostic tools, drug targets, and new vaccines to combat this pernicious disease.

## Supplementary Material

Supplemental Material
