# Supplementary material for: Quantitative proteomics reveals that dormancy-related proteins mediate the attenuation in mycobacterium strains
Source: Virulence. 2021 Oct 11;12(1):2228–46. doi: 10.1080/21505594.2021.1965703 (PMC8923072; doi:10.1080/21505594.2021.1965703)

Figure S1. Fraction and combination of TMT labeling peptides from the log-phase. (a) Liquid-phase separation diagram of labeled peptide mixtures. (b) The combination of separated fractions.

Figure S2. Large-scale quantitative proteomics for H37Rv, H37Ra, and BCG in log-phase by biological duplication. (a) The Gaussian fitting curve of the $\log_2$ ratio of the intensities of technical replicates. Red and green curves represent the Gaussian and experimental fitting curves, respectively. (b) Scatter plots and Spearman's correlation coefficients for proteome profiling of the four samples. The *x* and *y* axes represent the $\log_2$-transformed protein intensities in each two-sample comparison.

Figure S3. The fraction and combination of TMT labeling peptides from stationary-phase. (a) Liquid-phase separation diagram of labeled peptide mixtures. (b) The combination of separated fractions.

Figure S4. Large-scale quantitative proteomics for H37Rv, H37Ra, and BCG with different virulence in the stationary-phase. (a) The Gaussian fitting curve of the $\log_2$ ratio of the intensities of technical replicates. Red and blue curves represent the Gaussian and experimental fitting curves, respectively. (b) Scatter plots and Spearman's correlation coefficients for proteome profiling of ten samples. The x and y axes represent the $\log_2$-transformed protein intensities in each two-sample comparison.

Figure 5. Comparison of identified H37Rv proteins from the log- and stationary-phase. (a) Venn diagram of H37Rv proteins identified in the log- and stationary-phase. PPI analysis of the uniquely identified H37Rv proteins in the (b) log- and (c) stationary-phase with STRING. The area surrounding with the dotted line represents the functional protein cluster.

Figure S6. Functional analysis of consistently dysregulated proteins among H37Rv, H37Ra, and BCG. (a) Heat map analysis of the $\log_2$ ratio for the regulated proteins between H37Ra and H37Rv, BCG and H37Rv, and BCG and H37Ra. Red and green represent upregulated and downregulated proteins, respectively. (b) Functional categories of proteins identified in consistently upregulated and downregulated groups. Red and green represent upregulated and downregulated proteins, respectively.

Figure S7. PPI analysis of dysregulated proteins selected from log-phase. The interacting network diagram of the upregulated DEPs of Ra/Rv group (a), upregulated DEPs of BCG/Rv group (b), and upregulated DEPs of BCG/Ra group (c).

Figure S8. PPI analysis of the glyoxylate-related enzymes upregulated in BCG at the stationary-phase.

Figure S9. PPI analysis of the downregulated DEPs from the Ra/Rv group in the stationary-phase.

Fig. S1

a

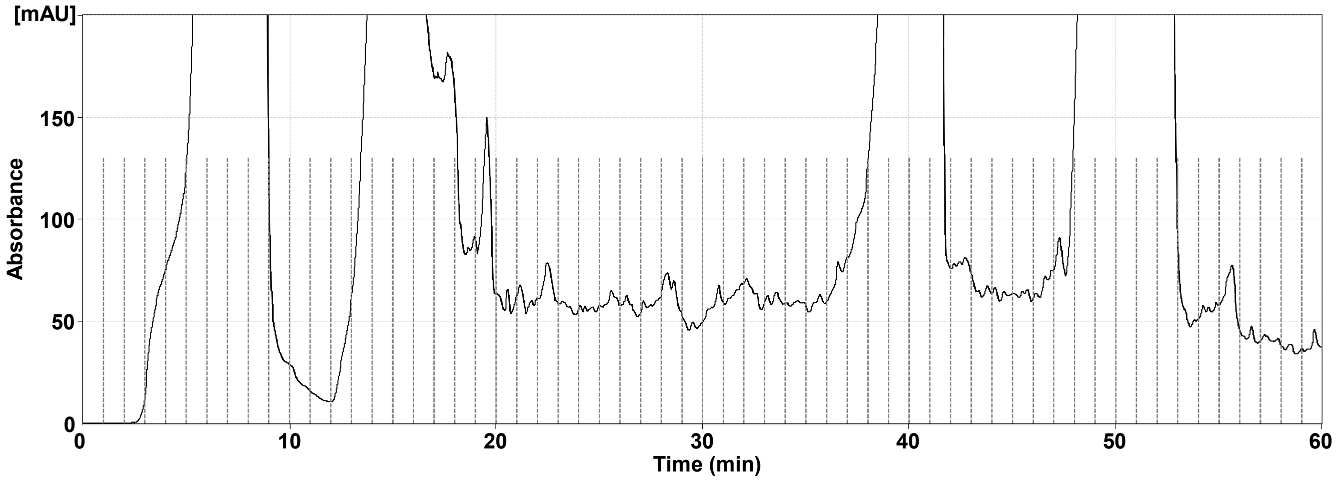

b

Table 1. The combination of separated fractions in log-phase.

No. of combined sample	1	2	3	4	5	6	7	8
No. of separated fraction	11	12	13	14	15	16	17	18
	19	20	21	22	23	24	25	26
	27	28	29	30	31	32	33	34
	35	36	37	38	39	40	41	42
	43	44	45	46	47	48	49	50
	51	52	53	54	55	56	57	58
	59	60						

Fig. S2

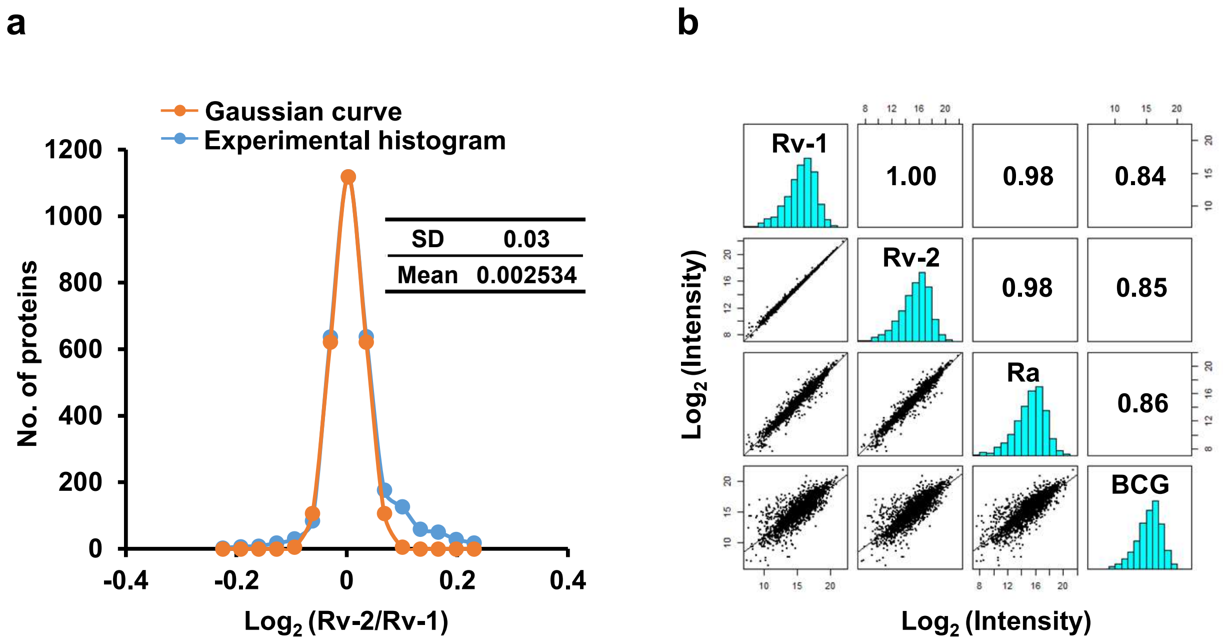
a
Gaussian curve
Experimental histogram
SD 0.03
Mean 0.002534
No. of proteins
1200
1000
800
600
400
200
0
-0.4
-0.2
0
0.2
0.4
Log2 (Rv-2/Rv-1)
b
Rv-1
1.00
0.98
0.84
Rv-2
0.98
0.85
Ra
0.86
BCG
Log2 (Intensity)
Log2 (Intensity)

Fig. S3

a

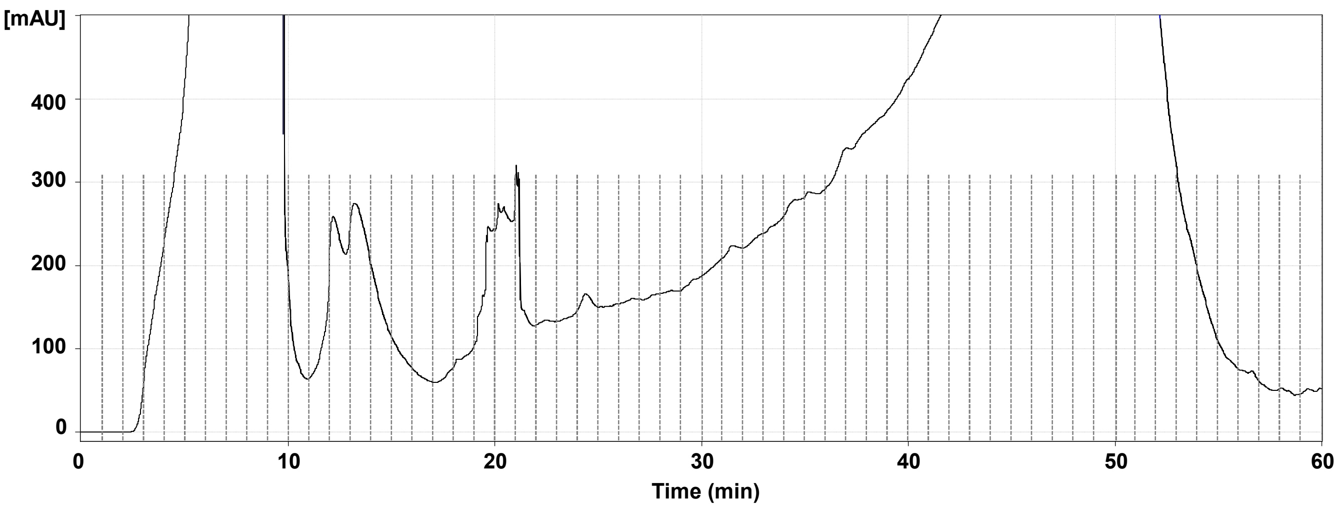

b

Table 2. The combination of separated fractions in stationary-phase.

No. of combined sample	1	2	3	4	5	6	7	8	9	10
No. of separated fraction	11	12	13	14	15	16	17	18	19	20
	21	22	23	24	25	26	27	28	29	30
	31	32	33	34	35	36	37	38	39	40
	41	42	43	44	45	46	47	48	49	50
	51	52	53	54	55	56	57	58	59	60

Fig. S4

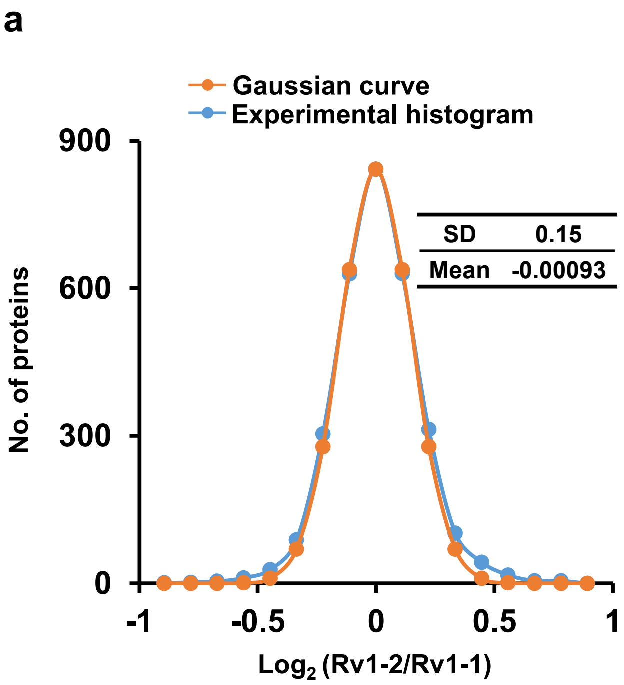
a
Gaussian curve
Experimental histogram
No. of proteins
900
600
300
0
-1
-0.5
0
0.5
1
Log2 (Rv1-2/Rv1-1)
SD 0.15
Mean -0.00093

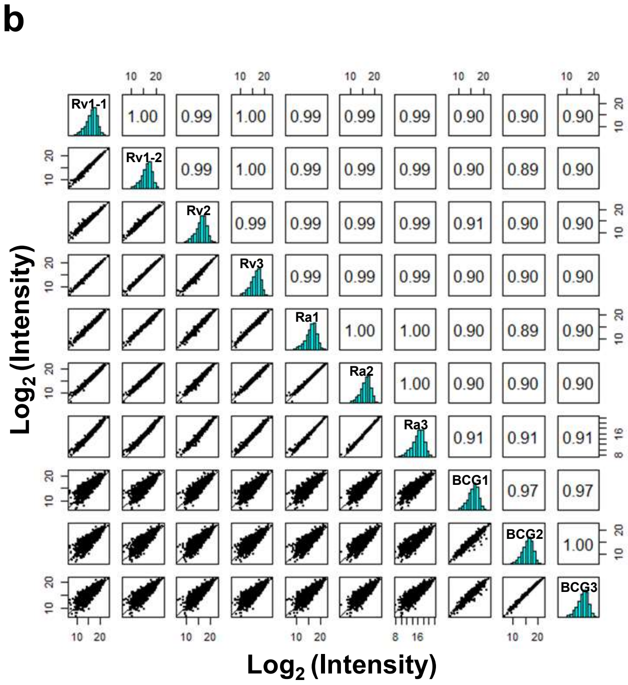
b
Rv1-1
Rv1-2
Rv2
Rv3
Ra1
Ra2
Ra3
BCG1
BCG2
BCG3
Log2 (Intensity)
Log2 (Intensity)

Fig. S5

a

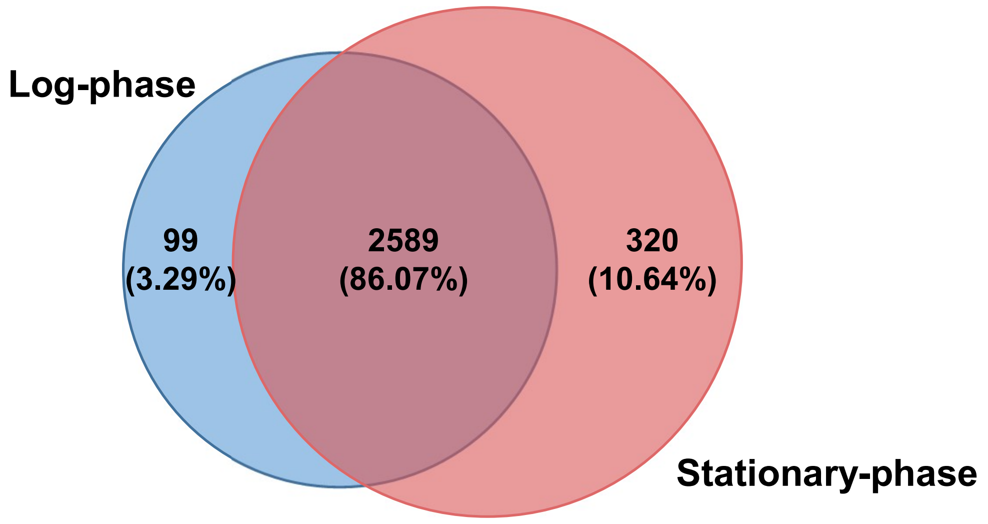

Fig. S5

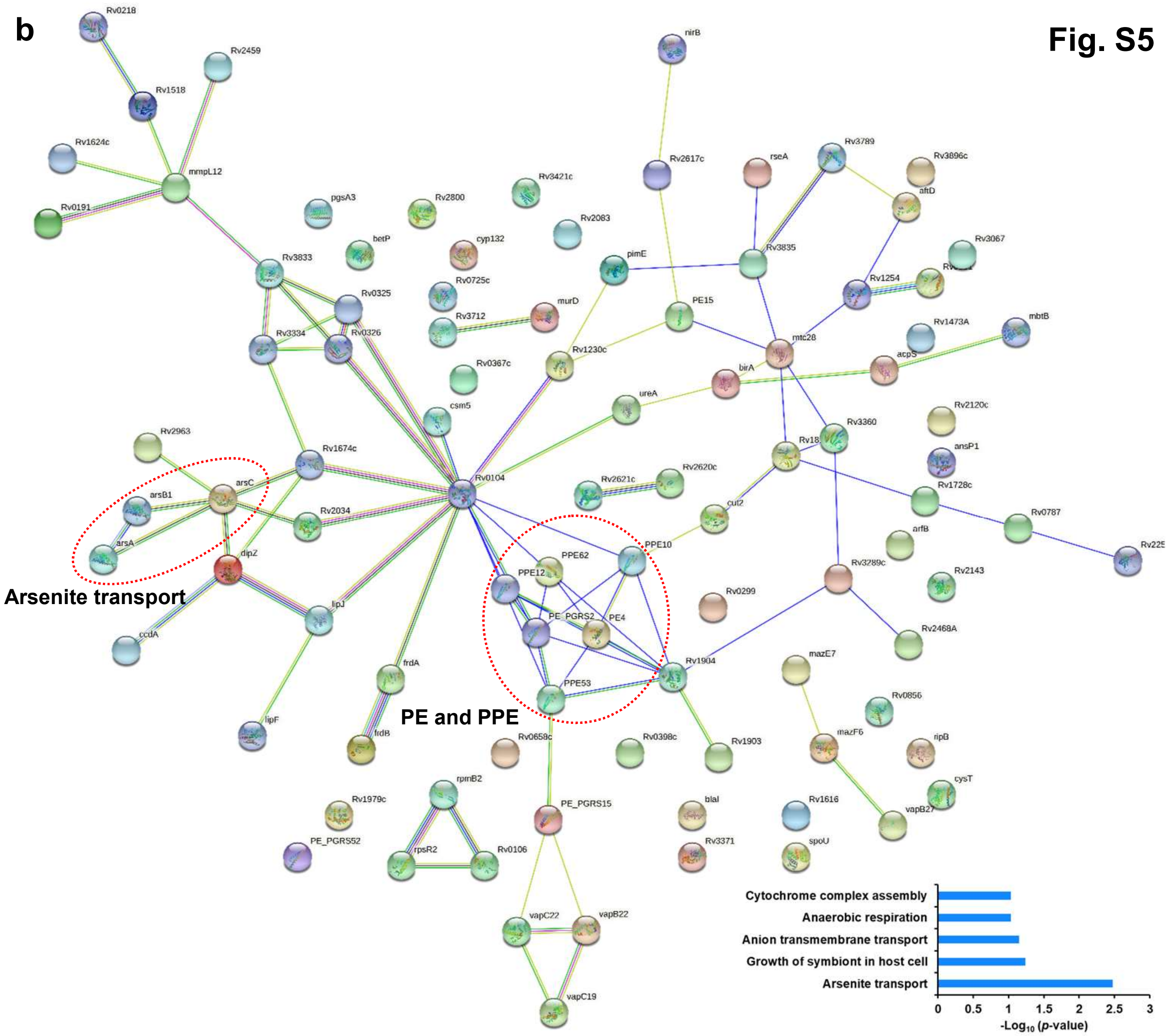
b
Arsenite transport
PE and PPE
Cytochrome complex assembly
Anaerobic respiration
Anion transmembrane transport
Growth of symbiont in host cell
Arsenite transport
0 0.5 1 1.5 2 2.5 3
$-Log_{10}$ (p-value)

c

Fig. S5

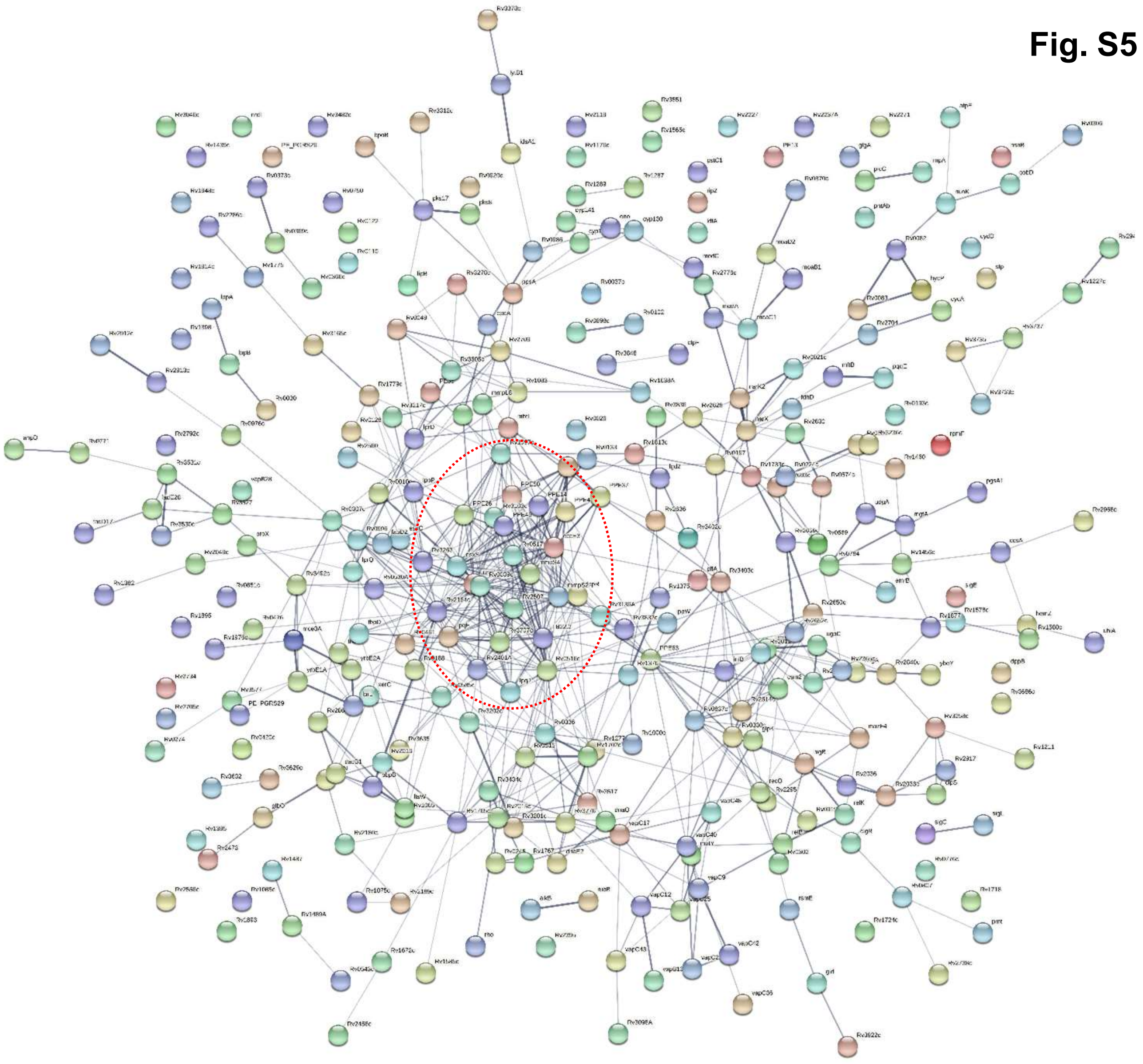

Fig. S6

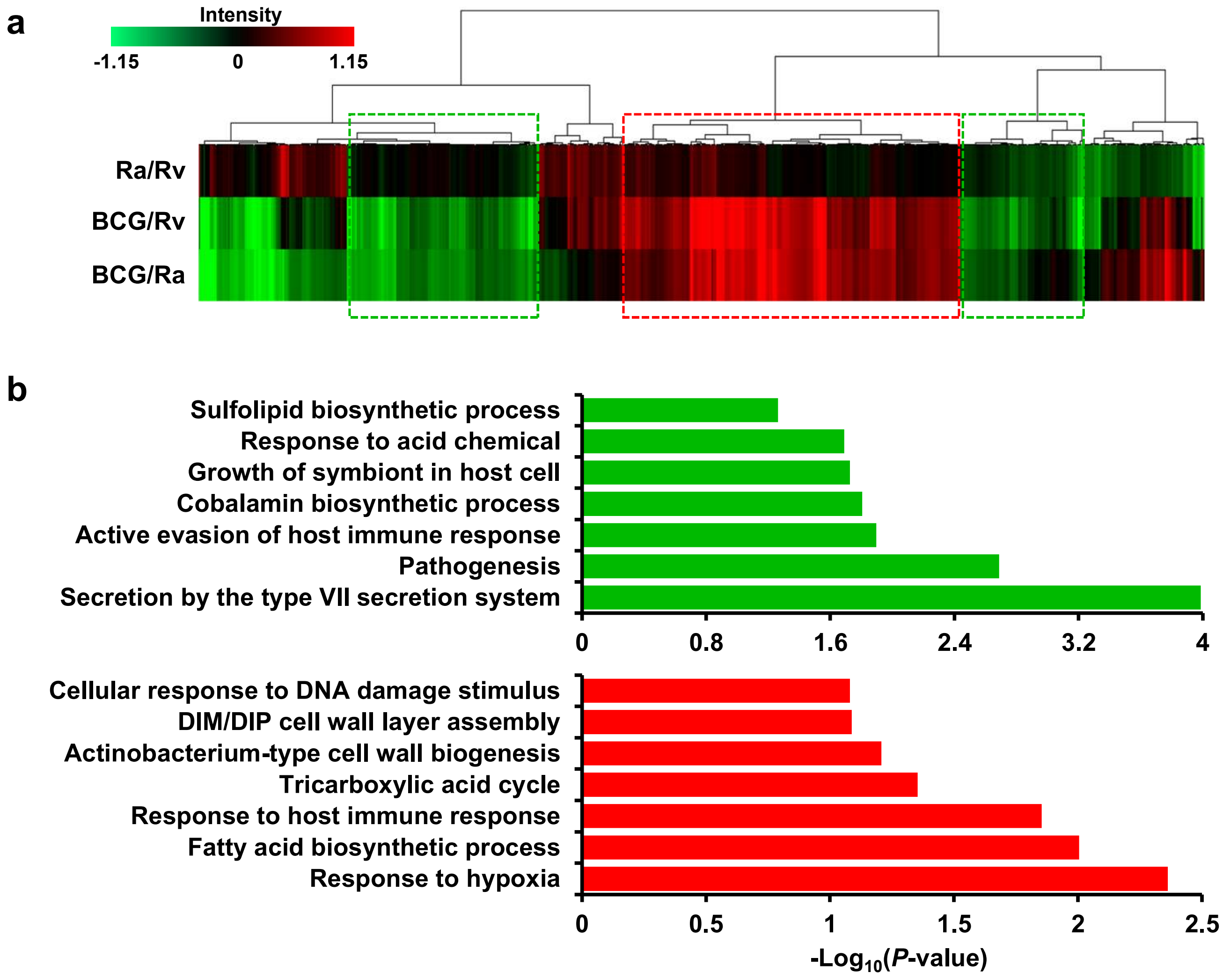

a
Intensity
-1.15
0
1.15
Ra/Rv
BCG/Rv
BCG/Ra
b
Sulfolipid biosynthetic process
Response to acid chemical
Growth of symbiont in host cell
Cobalamin biosynthetic process
Active evasion of host immune response
Pathogenesis
Secretion by the type VII secretion system
0
0.8
1.6
2.4
3.2
4
Cellular response to DNA damage stimulus
DIM/DIP cell wall layer assembly
Actinobacterium-type cell wall biogenesis
Tricarboxylic acid cycle
Response to host immune response
Fatty acid biosynthetic process
Response to hypoxia
0
0.5
1
1.5
2
2.5
-Log10(P-value)

a

Fig. S7

Up-regulated DEPs of Ra/Rv group in log-phase

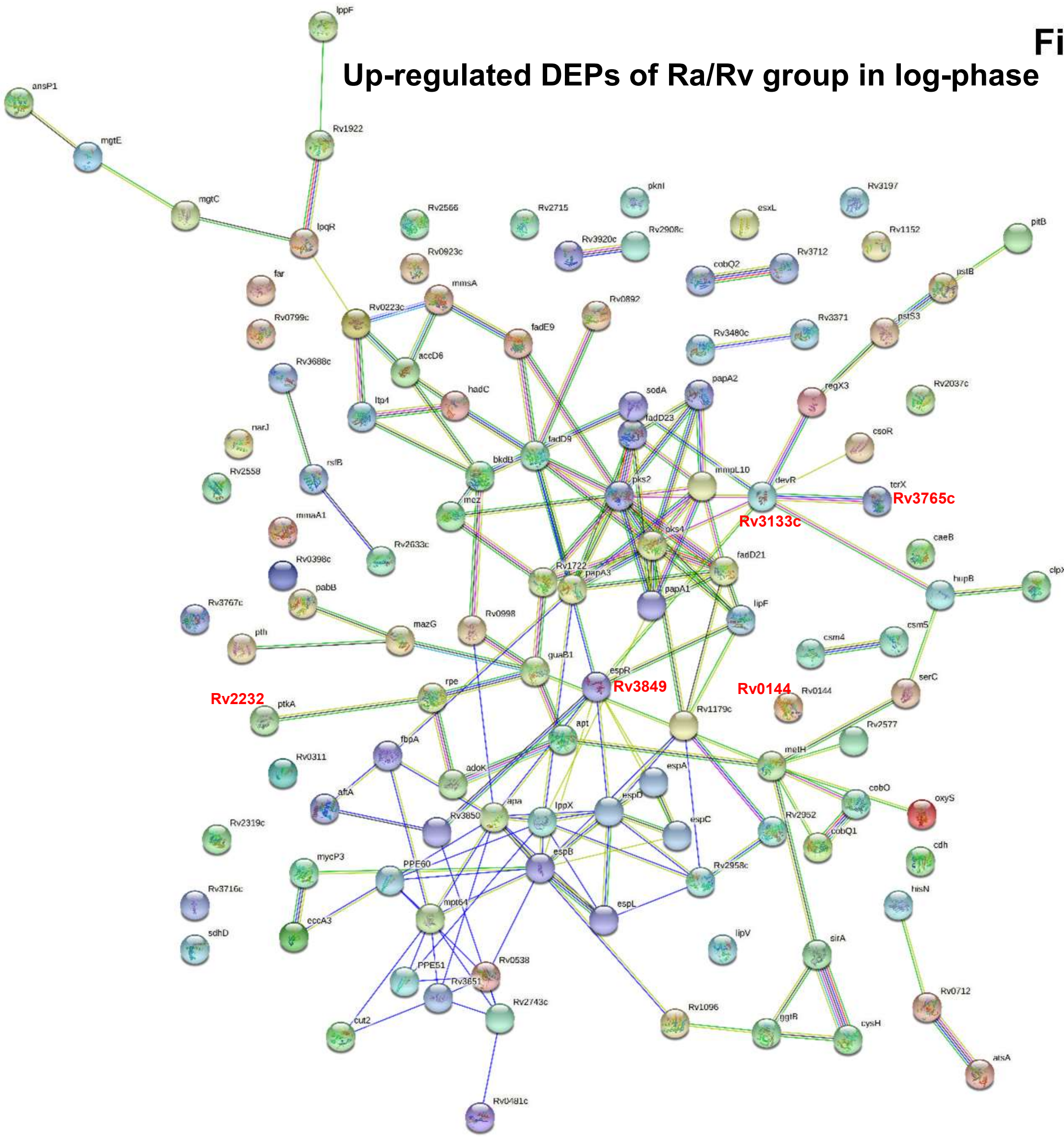

b

Fig. S7

Up-regulated DEPs of BCG/Rv group in log-phase

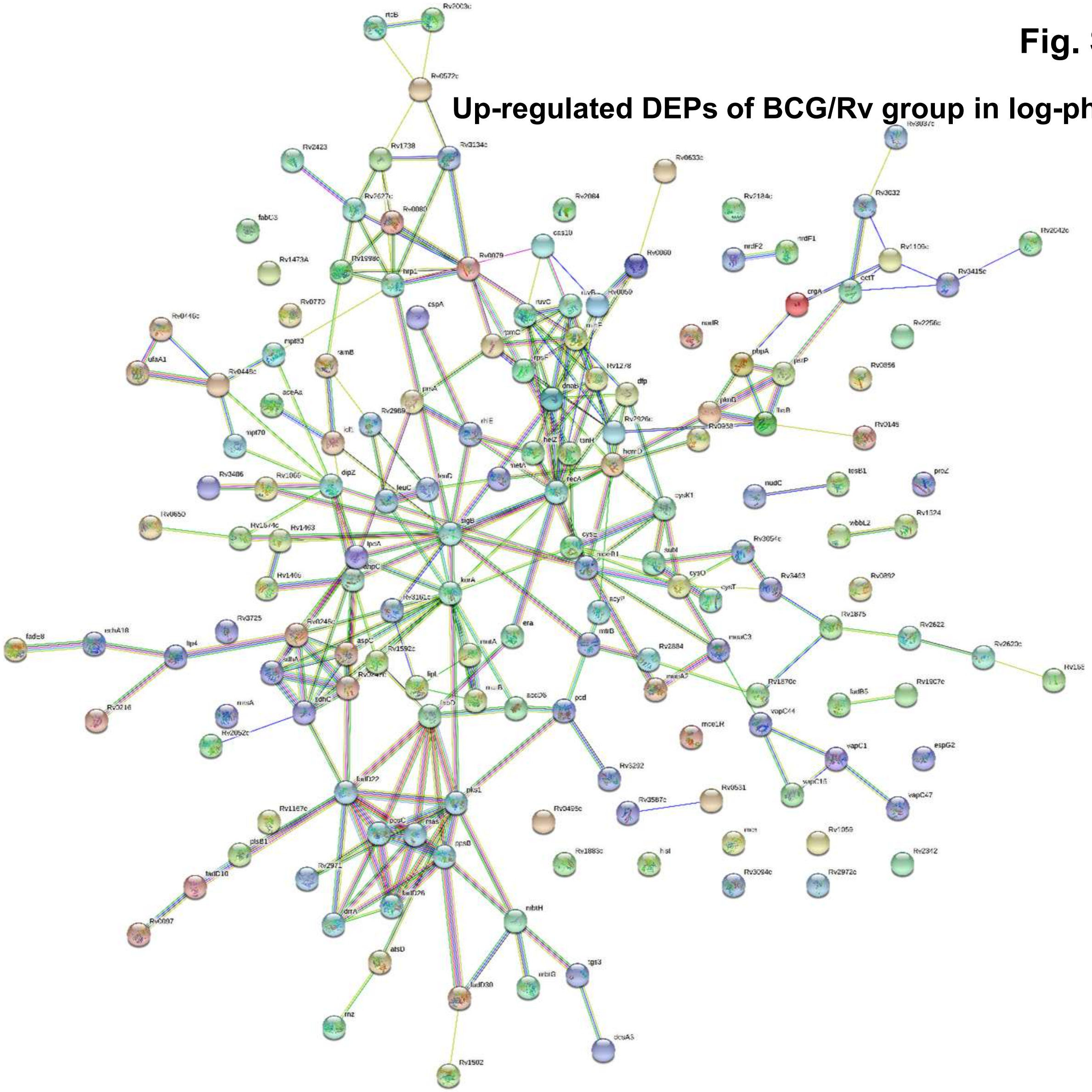

c

Fig. S7

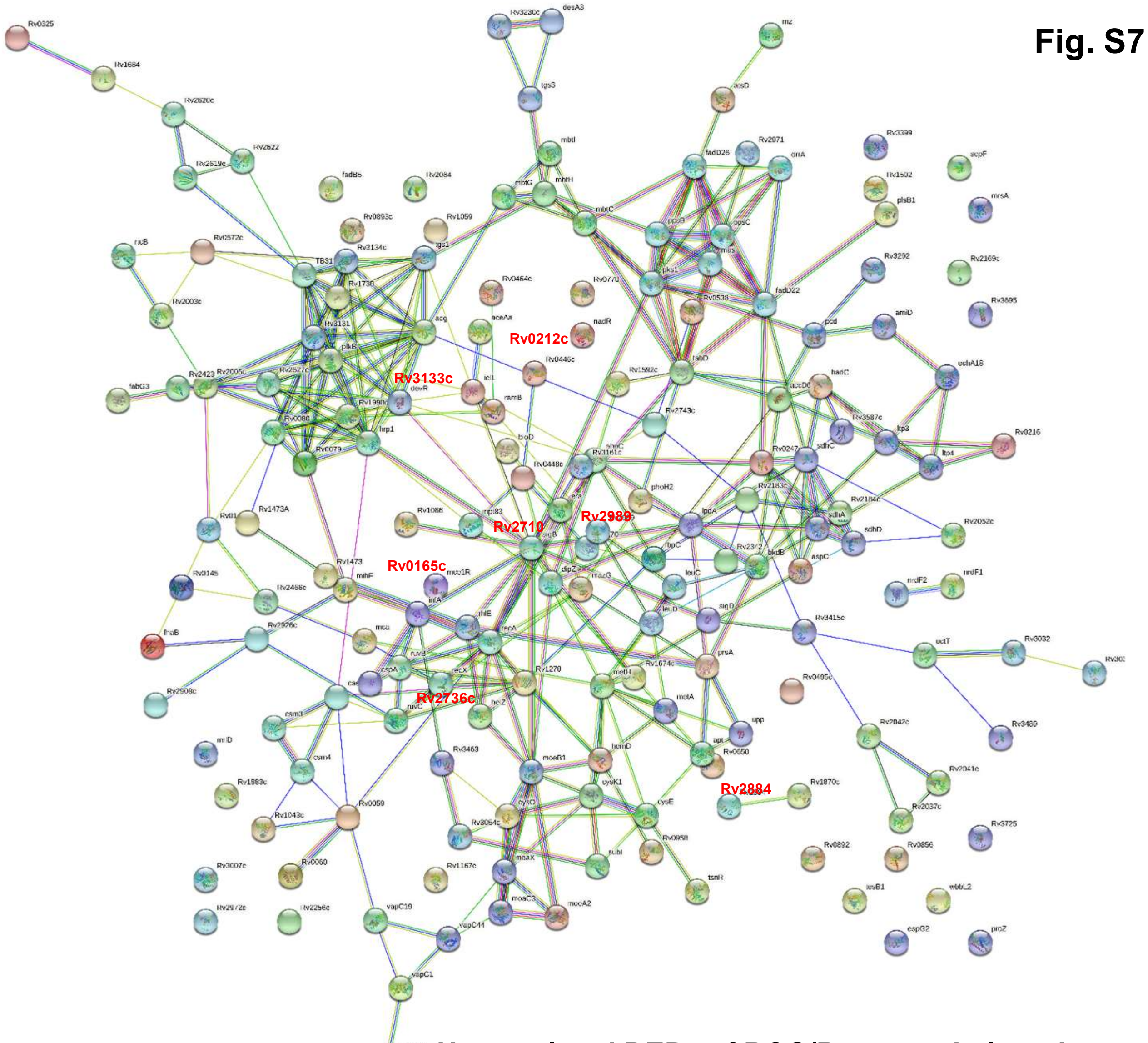

Up-regulated DEPs of BCG/Ra group in log-phase

Fig. S8

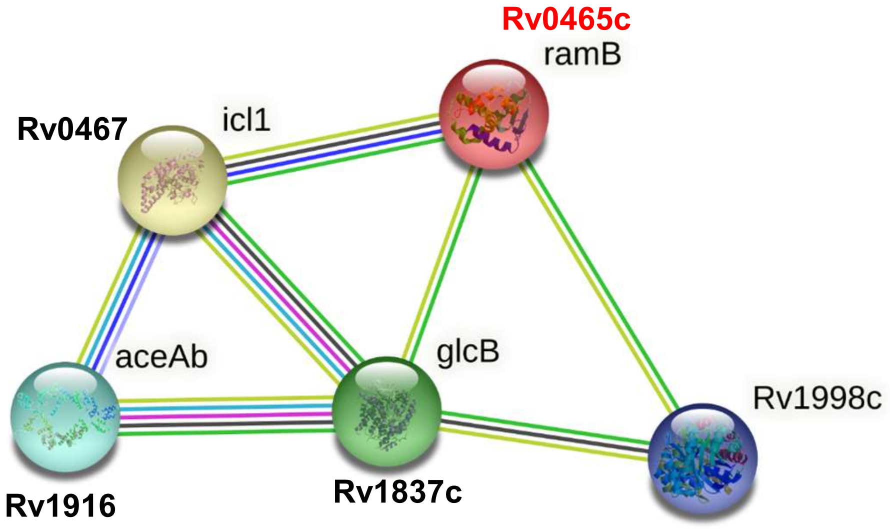
Rv0465c
ramB
Rv0467
icl1
aceAb
glcB
Rv1998c
Rv1916
Rv1837c

Fig. S9

Down-regulated DEPs from Ra/Rv group in stationary-phase

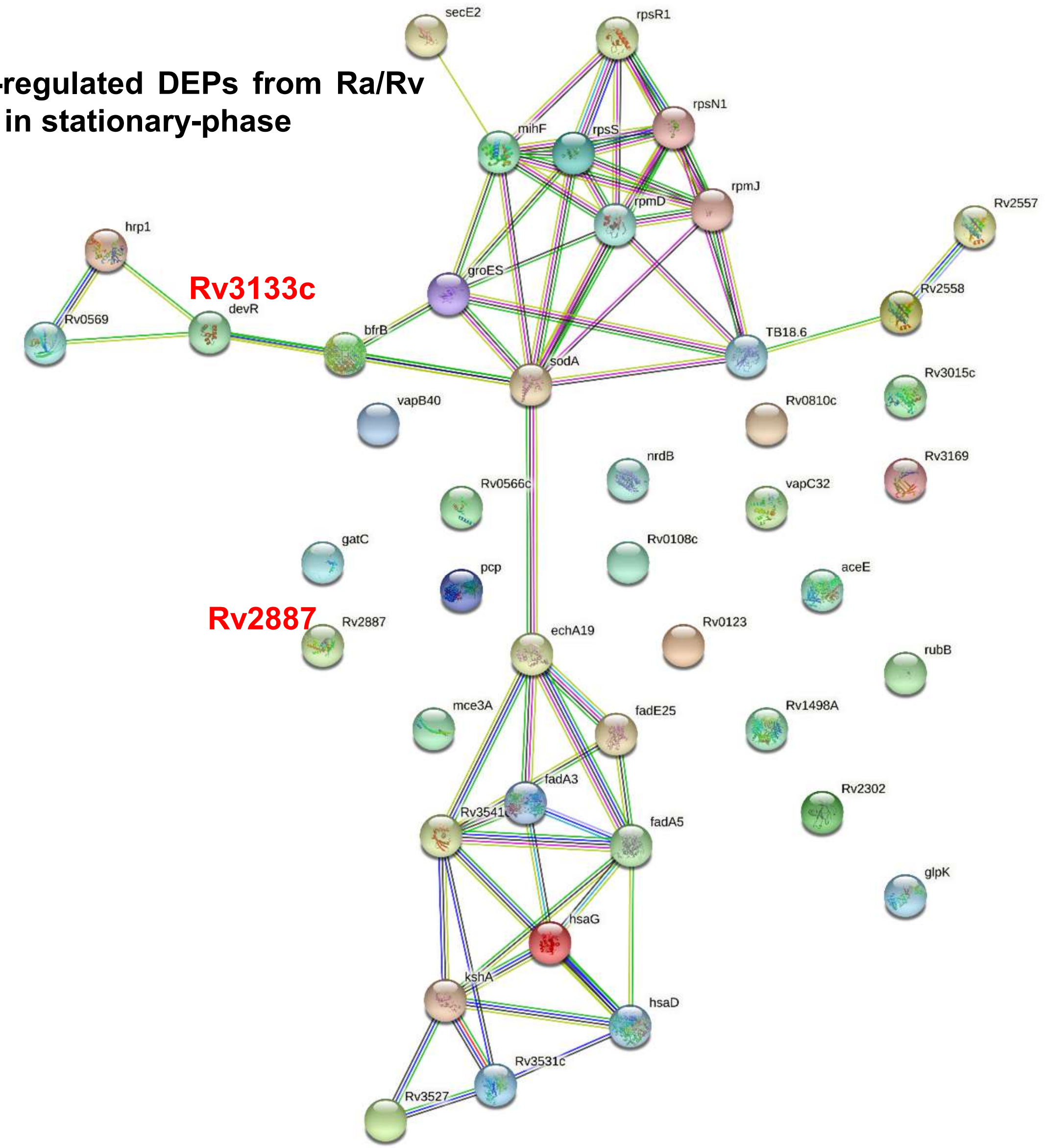

Supplement: Supplemental Material [file KVIR_A_1965703_SM0617.zip › supplementary/Supplementary figures-proof.pdf]
